# Comparison of Dutch healthy eating and healthy eating indexes and anthropometry in patients with major depression with health subjects: a case-control study

**DOI:** 10.3389/fnut.2024.1370562

**Published:** 2024-05-06

**Authors:** Melika Tohidi Nafe, Ariyo Movahedi, Abolghasem Djazayery

**Affiliations:** Department of Nutrition, Science and Research Branch, Islamic Azad University, Tehran, Iran

**Keywords:** major depression, Dutch Healthy Diet Index, healthy eating index 2015, PHQ-9, HEI

## Abstract

**Background:**

Diseases and disorders related to mental health are spreading like other chronic diseases all around the world. Considering the role of food in the prevention and treatment of these disorders, including major depression, investigating the relationship between different food patterns and this disorder is of particular importance. The aim of this study was to compare Dutch healthy eating and healthy eating indexes and anthropometry in patients with major depression with healthy individuals.

**Methods:**

In this case-control study, the final analysis was performed on 67 men and 111 women with an age range of 20–30 years. Height (cm), weight (kg), food frequency questionnaire (FFQ), physical activity (MET-min/week), demographic and PHQ-9 questionnaires were taken from all participants. In the following, all the food ingredients and their components were extracted and used to calculate HEI-2015 and DHD. Statistical analysis was performed using SPSS software with independent *t*-test, logistic regression and chi-square.

**Results:**

It was found that people with major depression in this study were mostly women and occupied. The average HEI-2015 in healthy people and those with major depression was 58 and 54.3, respectively. Also, the average DHD in these people was 60.5 and 55, respectively. HEI-2015 and DHD had a significant negative correlation with depression score (*r* = −0.16, *p*-value = 0.03) (*r* = −0.19, *p*-value = 0.01). Also, in the logistic regression model, before and even after adjusting confounders, HEI-2015 and DHD had a reduced odds ratio in people suffering from major depression. The two groups did not differ significantly in terms of the average factors of height, weight and body mass index (BMI).

**Conclusion:**

It seems that HEI2015 and DHD have a significant relationship in reducing major depression. However, due to the small number of studies in this regard, especially in the field of DHD, the need for more studies seems necessary.

## 1 Introduction

Depressive disorders are one of the most important problems and concerns in the world (1). According to the World Health Organization, the second main cause of illness-related costs in 2020 was depression. In general, depressive disorders have affected about 350 million people of different ages (2). In some studies, the prevalence of depression in Iran has been stated as 8.2–43.3%, depending on the target groups studied, and a major part of this difference is related to age and the type of method used to measure depression (3, 4). Many factors are involved in depression. One of these factors is gender. As it has been stated that its prevalence in women is 2 times that of men (5). Another risk factor is the family history of depression, and it is actually a type of genetic risk factor (6). Another risk factor is alcohol and nicotine abuse (7, 8). Chronic diseases are another risk factor. It has been suggested that diseases such as diabetes, cardiovascular disease, infertility and some other diseases can be related to depression (9, 10). Age is another risk factor, and the highest prevalence of depression has been reported between 60 and 64 years and related to chronic diseases (11). Some other risk factors include marital status (12), bad events in life (13), low income (14), low education level (6), weight gain and obesity (15), and food intake (16).

Today, with the progress of various sciences, especially molecular sciences, the relationship of many non-infectious diseases with diet has been noticed. Diet is considered as a factor of prevention and even treatment of diseases. The relationship between food intake and depression has been investigated from different aspects. Some studies have investigated macronutrient and micronutrient intake in depression. For example, a prospective study with a follow-up of 8.5 years compared people with insufficient intake of more than four micronutrients compared to those with low intake of one micronutrient and its relationship with depression. It was found that people with insufficient intake of more than four micronutrients are more likely to suffer from depression by 37% (17). In a cross-sectional study, it was shown that a decrease in the intake of legumes, fruits and vegetables and an increase in the intake of sweets and refined sugars were associated with an increased risk of depression (18). Over time, by the creation of the opinion that people always receive a total of food and nutrients and these substances can have synergistic or reducing effects on each other, conducting studies in the form of food patterns became popular. One of the types of food patterns is the healthy eating index (HEI). Various studies have investigated the relationship between healthy eating index and depression. A case-control study in Iran obtained five dietary patterns using factor analysis. The results showed that following the HEI from the factor analysis was associated with a 39% reduction in depression (19). Also, in another cross-sectional study conducted in soldiers, it was shown that those who received the highest HEI-2010 had an 80% reduction in depression (20). Another study was conducted on overweight and obese women. In this study, in the final model and after adjusting for obesity, age, education, marital status, and caloric intake, HEI was not associated with depression (21).

Another dietary pattern that has been investigated is known as the Dutch Healthy Diet Index (DHD-Index). This dietary pattern was designed in 2012 based on the 2006 Dutch Healthy Diet Guidelines. This profile has 10 components including physical activity, vegetables, fruits and juices, fiber, fish, saturated and trans fatty acids, acidic foods and drinks, sodium and alcohol. Van lee and colleagues believe that individuals with high adherence to this pattern will have both high intakes of vitamins and minerals and a high nutrient density diet (16). The index was revised in 2015 and its components were changed to 15 components. These 15 components are vegetables, fruits, whole grains, legumes, nuts, dairy products, fish, tea, fat and oils, coffee, red meat, processed meat, sweetened beverages and juices, alcohol and salt (22). Studies that have examined the relationship between DHD-Index and depression are limited. One of these studies is a prospective study that stated that in the crude model, DHD-index and DASH were associated with a reduction in the risk of depression. After adjusting for socioeconomic factors, lifestyle and cardiovascular factors, only DHD-index was associated with a 17% reduction in depression (23). Also, a cross-sectional study in diabetic patients showed that in the entire diabetic population, the highest quartile of receiving DHD is associated with a reduction in the risk of depression (24). While the DHD-Index was established using Dutch dietary standards, its core principles apply to a wide range of populations around the world. The index highlights foods and dietary behaviors that are universally acknowledged to be beneficial for health, such as eating fruits, vegetables, and whole grains and avoiding excessive saturated fats and sugar (16). As a result, the DHD-Index can be an effective tool for evaluating dietary patterns and their connections with health outcomes across groups, and it provides a standardized framework for assessing adherence to these key dietary principles across different cultural contexts, including Iran.

The greatest incidence of depression is seen in the 20–30 age group in Iranian population (25). The results of existing studies on the relationship between HEI2015, BMI and DHD with depression are contradictory and, in some studies, a significant relationship has been observed, while in others no relationship has been observed. On the other hand, the number of studies in some fields is insufficient and the need for more studies is felt. Therefore, we decided to compare Dutch healthy eating and healthy eating indexes and anthropometry in patients with major depression ranging from 20 to 30 years old with healthy people.

## 2 Materials and methods

### 2.1 Study population

This research was conducted in Mehregan Hospital, Tehran, and participants were 178 totally (including 67 men and 111 women) with age of 20–30 years from the main centers for mental disorders [All 105 patients consumed antidepressant and in questionnaire (PHQ-9), it was definitely determined that all healthy people did not have depression and tendency to it]. Sampling method was simple and available. All people who had the conditions to enter the study and were willing to cooperate were included in the project. The exclusion criteria were: The presence of diseases affecting reception such as tumors, and kidney problems, following weight loss diets, taking drugs that affect appetite, and completing < 70% of questions or refusing to continue questioning. In order to keep all information confidential, people were asked not to write their name and surname on the information sheet, and personal information (name and surname and contact number) were collected in a separate notebook using only a code and note. This research was reviewed by the medical ethics committee of Islamic Azad University and approved under the code of ethics IR.IAU.SRB.REC.1401.151.

### 2.2 Anthropometric measurements

Anthropometric indices, including height and weight, were measured according to a standard protocol. Weight was measured and recorded with minimal clothing and without shoes using a scale with an accuracy of 100 grams. The height of people was measured using a tape measure while standing next to the wall without shoes while the shoulders were in normal conditions with an accuracy of 0.1 cm. The body mass index (BMI) of the subjects was calculated by dividing the weight by the square of the height (kg/m^2^) of each person.

### 2.3 Dietary assessments

Nutritional information of people was collected face-to-face using a valid and reliable 147-item food frequency questionnaire (FFQ) (25, 26). People were asked to state their consumption of each of the 147 food items in the questionnaire in terms of day, week, month, and year. Finally, these amounts were converted into daily intakes in grams. A combination of the American food table was used to extract all the micro and macro nutrients available for each person (27). For mixed foods (such as pizza) the nutrients were calculated based on the sum of the nutrients of the food items that make up that food.

### 2.4 Calculation of HEI-2015

The calculation of this index was done in such a way that the data of the people were entered into the software and the macronutrients and micronutrients received by all the people participating in the study were calculated. HEI scoring was done based on previous studies (28).

### 2.5 Calculation of DHD-index

Ten components are required to calculate this index. Each of the components is scored from 0 to 10 and finally, with the sum of all components, the score range of this index is from 0 to 100 points. The components include physical activity, vegetables, fruits and juices, fiber, fish (even capsules containing fish oil), saturated fatty acids, trans fatty acids, acidic foods and drinks, sodium and alcohol (16).

### 2.6 Measuring depression

Major depression was measured in all subjects based on the PHQ-9 questionnaire. This questionnaire was designed and validated in 2001 by Robert Kroenke and his colleagues at Columbia University (29). The validity and reliability of this questionnaire has also been measured in Iran (30, 31). This questionnaire contains nine questions with a Likert scale. Answers to these questions are scored from 0 (in everything) to 3 (almost every day). Finally, each person received 0 to 27 points. A score above 20 was classified as severe depression, a score of 15–19 as moderately severe depression, a score of 10–14 as moderate depression, a score of 5–9 as mild depression, and a score of 0–4 as no depression.

### 2.7 Sample size

The sample size in the present study includes people living in Tehran. People were randomly selected. The sample size was calculated using G-power software version 3.1.9.7 (32) with settings for Linear Bivariate Regression studies and Correlation studies: Bivariate model as follows, and the highest number calculated in the above two cases is equal to 166 people. It is that by taking into account 20% probability of spillage, a total of 188 people were considered as a sample, who were randomly selected from among the volunteers who filled out the questionnaires. Finally, 80 people have been considered for each group, and considering the 10% reduction, 90 people have been considered for each group.

### 2.8 Statistical analysis

All data were analyzed using SPSS version 26. In the present study, correlation between DHD, HEI and major depression indexes was investigated. Shapiro-Wilk test and histogram curve were used to check the normality of the variables, and Chi-square test was used to determine the relationship between qualitative independent variables. Pearson's correlation was used to examine the relationship between quantitative variables. *T*-test was used to compare the data. Also, logistic regression was used in order to measure correlation and adjusting confounders. *P*-value < 0.05 was considered as significant.

## 3 Result

Determination and comparison of demographic characteristics, HEI, DHD, and anthropometric measurements between healthy people and patients with major depression.

One hundred and eighty-eight people were examined. A number of people were excluded from the study due to the defects of the questionnaires and non-cooperation, and thus, the final analyzes were performed on 178 people. In chi-square analysis, it was found that healthy people and people with depression in terms of gender (*P* = 0.04), employment status (*P* = 0.019), family history of depression (*P* = 0.02) and history of depression (*P* = 0.001) had a significant difference with each other. A higher percentage of depression was observed in the working group compared to students or unemployed people; However, in the comparison of the two groups, no difference was observed in terms of marital status, education, smoking, alcohol consumption, special diet, or omega-3 consumption (Table 1). In the *t*-test analysis, it was found that healthy people have a higher average HEI index than the depressed group. This difference was statistically significant (*P* = 0.004) (Table 1). More than that, the average DHD index in healthy and sick people was 60.5 and 55.0, respectively, and this difference was statistically significant (*P* = 0.004) (Table 1). Plus, the two groups were not significantly different in terms of weight, height and BMI. The average BMI in the healthy and depressed groups was calculated as 25.4 and 24.6 kg/m^2^, respectively (Table 1).

**Table 1:** Determination and comparison of demographic characteristics between healthy people and patients with major depression.

		**Healthy participants *n* = 73 (%)**	**Participants with depression *n* = 105 (%)**	*p*-value^*^
Gender	Man	34 (46.6)	33 (31.4)	
	Woman	39 (53.4)	72 (68.6)	
Marital status	Single	31 (42.5)	59 (56.2)	
	Married	41 (56.2)	43 (41)	
	Divorced	1 (1.3)	3 (2.8)	
Education	High school	0 (0)	5 (4.8)	0.14
	Diploma and associate	5 (6.8)	9 (8.6)	
	Bachelor	30 (41.1)	53 (50.5)	
	Master	31 (42.5)	31 (29.5)	
	PhD	7 (9.6)	7 (6.6)	
Occupation	Unemployed	2 (2.7)	10 (9.5)	0.01
	student	15 (20.5)	35 (33.3)	
	employed	56 (76.8)	60 (57.2)	
Smoking	No	62 (84.9)	88 (83.8)	0.84
	Yes	11 (15.1)	17 (16.2)	
Drinking alcohol	No	62 (84.9)	90 (85.7)	0.14
	Less than half a glass per week	4 (5.5)	9 (8.6)	
	Half to 1.5 glasses per week	5 (6.8)	1 (1.0)	
	More than 1.5 glasses per week	2 (2.8)	5 (4.7)	
Omega-3	No	62 (84.9)	91 (86.7)	0.74
	Yes	11 (15.1)	14 (13.3)	
Being on a diet	No	66 (90.4)	90 (85.7)	0.34
	Yes	7 (9.6)	15 (14.3)	
Family history of depression	No	60 (82.2)	70 (66.7)	0.02
	Yes	13 (17.8)	35 (33.3)	
History of depression	No	70 (95.9)	73 (69.5)	0.001
	Yes	3 (4.1)	32 (30.5)	
HEI (achieved score)		58.0 ± 8.0	54.3 ± 8.6	**0.004**
DHD (achieved score)		60.5 ± 7.4	55.0 ± 7.8	**0.007**
Weight (kg)		169 ± 9.9	167.6 ± 8.8	0.35
Height (cm)		73.2 ± 17.2	69.5 ± 15.4	0.16
BMI (kg/m^2^)		24.6 ± 4.2	24.6 ± 4.2	0.22

^*^Obtained from chi-square and independent *t*-test.

HEI, Healthy Eating Index; DHD, Dutch Healthy Diet; BMI, Body Mass Index. *p*-value less than 0.05 is considered to be statistically significant.

### 3.1 Determining the relationship between HEI and DHD index and depression score

In Pearson's correlation, it was found that there is a negative but very weak correlation and a significant relationship between HEI and depression score (*P* = 0.03, *r* = 0.16). Also, there was a negative but very weak correlation and a significant relationship between DHD index and depression score (*P* = 0.01, *r* = −0.19).

### 3.2 Determining the relationship between HEI and the incidence of major depression

Logistic regression was used to investigate the relationship between following HEI and the occurrence of major depression. In the crude models, model 1 and also model 2, the increase in compliance with HEI was related to the decrease in the risk of major depression (Table 2).

**Table 2:** Determining the relationship between HEI and the incidence of major depression.

	**CI (%)**	*P*-value^*^
Crude model	0.94 (0.91–0.97)	**0.005**
Model 1	0.95 (0.92–0.99)	**0.01**
Model 2	0.95 (0.92–0.99)	**0.02**

^*^Obtained from logistic regression.

Model 1: adjustment for gender, occupation, family history and history of depression.

Model 2: Model 1 with the addition of kilocalories and physical activity. *p*-value less than 0.05 is considered to be statistically significant.

### 3.3 Determining the relationship between DHD and major depression

Logistic regression was used to investigate the relationship between following DHD and the occurrence of major depression. In the crude models, model 1 as well as model 2, the increase in compliance with DHD was related to the decrease in the risk of major depression (Table 3).

**Table 3:** Determining the relationship between DHD and the incidence of major depression.

	**CI (%)**	P-value^*^
Crude model	0.95 (0.91–0.98)	**0.009**
Model 1	0.94 (0.90–0.98)	**0.01**
Model 2	0.94 (0.90–0.99)	**0.03**

^*^Obtained from logistic regression.

Model 1: adjustment for gender, occupation, family history and history of depression.

Model 2: Model 1 with the addition of kilocalories and physical activity. *p*-value less than 0.05 is considered to be statistically significant.

## 4 Discussion

In this study, it was found that healthy and depressed people had significant differences with each other in terms of gender, employment status, family history of depression, and history of depression. The results of the present study are in line with the results of some studies, such as a cross-sectional study conducted on 554 people which showed that the depression score differed significantly according to gender, and it was significantly higher in women aged 16–23 years and in women aged 60–89 years was significantly lower than men of the same age (33). Also, in the study of Ma et al., women constituted a higher percentage of depressed people (34). Also, Kuczmarski et al. showed in their study that women have a significantly higher depression score than men (35). Of course, the current results are in contrast with the results of some other studies, including Exebio and his colleagues. They observed that the depression score was higher in women, although it was not significant (36). In the present study, working people had the most depression, while in Suchomlinov's study, retired people had a significantly higher depression score than students and working people. Also, in the study of Lee et al., who compared working people, people who never had a job, retired people, and unemployed people, they observed that unemployed and retired people have more depression (37). The HEI profile was linked to a lower incidence of serious depression in logistic regression. Confounding factor correction did not change the significance of this connection. The present study's findings are consistent with those of other research, such as a study conducted by Lanuza et al. on 2031 adults over 60 years of age, which found that a 72% lower incidence of serious depression was connected with those who consumed the healthiest foods (38). Additionally, Sánchez-Villegas et al. found that those in the fifth quintile of HEI-2010 had a 23% lower incidence of depression than those in the first quintile in a prospective analysis with a 10-year follow-up of 15,093 participants. These correlations held true even after controlling for age, gender, BMI, smoking, physical activity, and energy consumption (39). Furthermore, Nouri and colleagues found that the only healthy eating patterns linked to a 39% lower risk of major depression were those that were consistent with marital status, education, family history of depression, occupation, smoking, sleep patterns during the day and at night, income, and food security in 510 healthy and depressed individuals aged 19–65 (19).

Studying the correlation of the Healthy Eating Index (HEI) in the Iranian population not only provides insights into dietary patterns and their impact on physical health but also sheds light on its relationship with mental health outcomes, particularly major depression. Several research studies have investigated this correlation, highlighting the importance of dietary quality in mental wellbeing. For instance, studies by Khakpouri et al. explored the association between HEI and cardiovascular risk factors in Iranian adults, revealing a significant inverse relationship between HEI scores and markers of cardiovascular disease, which are often comorbid with depression (40). Furthermore, investigations by Saneei et al. (41) assessed the HEI in relation to metabolic syndrome and diabetes risk in the Iranian population, conditions that have been linked to an increased risk of depression (42). Moreover, Chegini et al. examined the relationship between HEI and mental health outcomes among Iranian older adults, suggesting that adherence to a higher HEI was associated with a reduced risk of major depression (43). Overall, these studies collectively underscore the significance of the Healthy Eating Index as a valuable tool for assessing dietary quality and its impact on various health outcomes, including major depression, in the Iranian population.

In this study, it was found that healthy subjects have a higher average HEI index than the depressed group. Many existing studies refrained from expressing the average of this profile between healthy people and those suffering from major depression and expressed it in the tertiles of receiving food patterns or types of profiles under investigation, or expressed it in general in the entire population (20, 38, 42). And this made it difficult to draw conclusions in this hypothesis.

The Dutch Dietary Guidelines place a strong emphasis on the consumption of fruits, vegetables, whole grains, fish, nuts, and legumes, while restricting the intake of processed meats, sugar-filled beverages, and harmful fats. This is measured by the DHD-index. The association between major depression risk reduction and DHD-index adherence has been explained by a number of different processes. First and foremost, the DHD-index encourages a healthy diet rich in vital nutrients, such as vitamins, minerals, and omega-3 fatty acids, all of which are proven to be important for neurotransmitter activity and brain health (23). Furthermore, following the DHD-index is frequently linked to better lifestyle choices, such consistent exercise and abstaining from alcohol, both of which enhance mental health results. The negative link between major depressive disorder incidence and DHD-index adherence has been strongly supported by recent epidemiological studies, underscoring the role that dietary patterns play in promoting mental health (44).

In this study, it was found that the average DHD index in healthy people is significantly higher than that of people with depression. Our results are consistent with the only available study. Gianfredi et al., who studied 2,646 people in a cohort study, showed that the average DHD in healthy subjects was higher than in subjects with depression (84.5 vs. 79.6) and this difference was significant (23).

In this study, it was found that the two groups were not significantly different in terms of weight, height and BMI. The average BMI in this study was 25. Our results are in line with Suchomlinov et al. study which observed that there was a negative and significant correlation between height and depression score, although this relationship disappeared after adjusting for gender. Also, this negative relationship was significant only in students. No correlation was observed between BMI and depression score. BMI was not significantly different between the two groups (33). Also, in a study on 13,975 people, Ma and his colleagues observed that BMI values and waist-to-height ratio in people with depression compared to healthy people were 30.3 vs. 28.7 kg/m^2^ and 61.5 vs. 58.8, respectively and it was non-significant. However, in the regression models and in the final adjusted model (for age, sex, race, educational status, marital status, smoking, and alcohol consumption), underweight and obese individuals were associated with 79 and 55% odds of developing depression compared to individuals with normal BMI. But such a relationship was not observed in overweight people (34). Also, in a cross-sectional study, Hadi et al. investigated the relationship between abdominal volume profile as a predictor of the relationship between depression, anxiety and obesity. In this study, 307 overweight and obese people were investigated. The average BMI in healthy and depressed people was 32.5 vs. 33.5, which was not significant (45). Although our results are in conflict with the results of some studies, including Khan et al., in a study on 60 Pakistani people aged 18–60 years, they reported that the average BMI in healthy people and those with major depression was 0.22 vs. 24.6, respectively, and it was significant. However, the average height and weight between the two groups was not significant (46). The most important reason for the contradiction between the present study and other studies is the sample size and the age range of people.

In logistic regression, HEI index was found to be associated with reduced risk of major depression. This relationship remained significant after adjusting for confounding factors. The results of the present study are in line with the results of some studies, including Lanuza et al. in a study on 2031 people over 60 years of age. They observed that people with the healthiest food intake were associated with a 72% reduction in the risk of major depression (38). Also, in a prospective study with 10-year follow-up of 15,093 people, Sánchez-Villegas et al. observed that people in the fifth quintile of HEI-2010 had a 23% reduced risk of depression compared to people in the first quintile. After adjusting for age, sex, BMI, smoking, physical activity and energy intake, these relationships remained significant (39). Also, in a study of 1,118 men and women aged 30–64, Kuczmarski et al. observed that HEI was inversely associated with depressive symptoms. For every one unit increase in HEI score, the chance of depression symptoms decreased by 0.98. In the linear regression model, after adjusting for gender, age and income, significant relationships remained (35).

In logistic regression, it was found that DHD index was associated with a reduced risk of major depression. This relationship remained significant even after adjustment for confounding factors. Studies examining the relationship between DHD and major depression are very limited. The findings of the present study are consistent with the findings of existing studies. Including Gianfredi et al. in a prospective study they conducted on 2,646 Dutch men and women in 2021 observed that in the primary model DHD is associated with a reduced risk of depression. Also, after adjusting for socioeconomic factors, lifestyle and cardiovascular factors, only DHD-index was associated with a 17% reduction in depression (23). Also, in a study published in 2020, Vogtschmidt et al. examined the relationship between DHD and depressive symptoms in healthy and diabetic individuals. In the results, it was found that in the entire diabetic population, the highest quartile of receiving DHD was associated with a reduction in the risk of depression (24).

The strength of this study is the comparison of HEI and DHD index for the first time, and the inevitable weakness of this study is the possibility of errors in filling out the questionnaires by people suffering from depression and mental problems. In our study, we focused on depression, but these people may also have other disorders that may influence the results in terms of nutrition and appetite, as well as how they respond to FFQ.

## 5 Conclusion

The results of this study showed that people with higher HEI and DHD indexes have lower depression scores. According to these results and the findings of previous studies, it seems that food observances and abstinences, alcohol consumption and reduced physical activity can be an important factor in causing depression. Although more complete and comprehensive studies are needed in this field. We suggest that prospective research be conducted in nutrition and psychology. Each group collects the necessary data, and in the end, collaborative and extensive investigations are conducted on the association between intakes and dietary patterns and depression, stress, and anxiety. Health clinics and schools appear to be the greatest places to project this duty.

## Data availability statement

The original contributions presented in the study are included in the article/supplementary material, further inquiries can be directed to the corresponding author.

## Ethics statement

This research was reviewed by the Medical Ethics Committee of Islamic Azad University and approved under the code of ethics IR.IAU.SRB.REC.1401.151. The studies were conducted in accordance with the local legislation and institutional requirements. The participants provided their written informed consent to participate in this study. Written informed consent was obtained from the individual(s) for the publication of any potentially identifiable images or data included in this article.

## Author contributions

MT: Conceptualization, Data curation, Formal analysis, Funding acquisition, Investigation, Methodology, Project administration, Resources, Software, Supervision, Validation, Visualization, Writing—original draft, Writing—review & editing. AM: Investigation, Supervision, Validation, Writing—review & editing. AD: Validation, Writing—review & editing.
